# Ecological Trade-Offs Between Mangrove Expansion and Waterbird Diversity: Guild-Specific Responses to Pond-to-Mangrove Restoration

**DOI:** 10.3390/ani16020299

**Published:** 2026-01-19

**Authors:** Cheng Cheng, Miaomiao He, Cairong Zhong, Xiaobo Lv, Haijie Yang, Wenqing Wang

**Affiliations:** 1 Hainan Academy of Forestry (Hainan Academy of Mangrove), Haikou 571100, China; cc401@163.com (C.C.);; 2 College of Life Sciences, Hainan Normal University, Haikou 571158, China; 3 Key Laboratory of the Ministry of Education for Coastal and Wetland Ecosystems, College of the Environment & Ecology, Xiamen University, Xiamen 361102, China; 4 National Observation and Research Station for the Taiwan Strait Marine Ecosystem, Xiamen University, Zhangzhou 363000, China; 5 Haikou Wetland Protection Engineering Technology Research and Development Center, Haikou 571100, China

**Keywords:** pond-to-mangrove restoration, mangrove, waterbird diversity, habitat trade-off, shorebirds

## Abstract

Coastal pond-to-mangrove restoration has become a prominent Nature-based Solution, yet its short-term ecological effects on waterbird communities remain unclear. We assessed taxonomic, functional, and compositional responses of waterbirds to large-scale restoration in Bamen Bay, Hainan Island, using BACI-style comparisons between restored and unrestored aquaculture ponds in 2021 and 2023. Restored areas exhibited higher taxonomic α diversity and functional richness (*p* < 0.001), coinciding with rapid habitat diversification following hydrological reconnection. Species richness (*p* < 0.001), Shannon diversity (*p* < 0.01), and functional richness (*p* < 0.01) were consistently higher in restored areas than in aquaculture ponds. In contrast, β diversity patterns diverged between habitats: restored areas remained relatively stable, whereas aquaculture ponds showed greater between-year compositional change (*p* < 0.05). Guild-specific responses revealed contrasting patterns: herons showed higher diversity in restored habitats (*p* < 0.05), whereas shorebirds exhibited no significant changes (*p* > 0.05), consistent with their dependence on open mudflats that were only partially retained. Although no significant declines were detected, functional richness tended to be lower in 2023 (*p* > 0.05), and ongoing mudflat loss suggests potential long-term risks for mudflat specialists, warranting extended monitoring. Taken together, our findings suggest that effective pond-to-mangrove restoration in Bamen Bay should balance mangrove expansion with the retention of tidal flats and managed shallow-water habitats to support diverse waterbird assemblages.

## Simple Summary

Mangrove restoration is rapidly expanding across coastal regions, yet its benefits and risks for waterbirds remain uncertain. In this study, we examined how waterbird communities responded to pond-to-mangrove restoration in Bamen Bay, Hainan Island, by comparing restored mangrove ponds with nearby aquaculture ponds. We found that restoration quickly increased the number of bird species and supported a wider range of ecological functions. Herons adapted well to the new habitats created by tidal reconnection and young mangrove growth. Shorebirds, which depend on open mudflats, showed no clear improvement in this short-term comparison and may be more sensitive if mangroves continue to expand, a possibility that will require longer-term monitoring. Our study highlights that mangrove restoration can enhance biodiversity but also reduce the habitats needed by mudflat specialists. Successful restoration should therefore balance mangrove expansion with the conservation of open tidal flats to support diverse waterbird communities.

## 1. Introduction

Mangrove ecosystems, situated at the land–sea interface in tropical and subtropical regions, deliver disproportionate ecological value relative to their global extent. They function as major carbon sinks, buffer coasts from storms and erosion, support nutrient cycling, and provide essential habitat for diverse fish, invertebrate, and waterbird communities [1,2,3]. Along the East Asian–Australasian Flyway (EAAF), migratory waterbirds depend on a network of coastal wetlands for stopover and wintering. Mangroves contribute to this network by providing roosting habitat and, together with adjacent tidal flats and shallow waters, offering complementary foraging opportunities for different waterbird guilds [4,5,6]. Despite their importance, mangroves have continued to decline globally. Since the mid-twentieth century, more than one-third of mangrove cover has been lost, with the steepest losses reported in East and Southeast Asia, where coastal development, aquaculture expansion and hydrological modification remain widespread [7,8,9]. In southern China, rice expansion accounted for 48% of mangrove loss (>210 km^2^) from the 1950s to 2010, and Myanmar’s Ayeyarwady Delta lost 44% (~940 km^2^) of its mangroves between 1989 and 2000 [9,10,11]. This rapid habitat loss not only undermines ecological functioning but also reduces the availability of essential stopover and wintering sites for EAAF waterbirds, many of which are already experiencing steep population declines [6,12]. As a result, restoring degraded mangrove ecosystems has become a central component of global Nature-based Solutions aimed at safeguarding coastal resilience, mitigating biodiversity loss, and recovering critical waterbird habitats [13,14].

**Figure 1:**
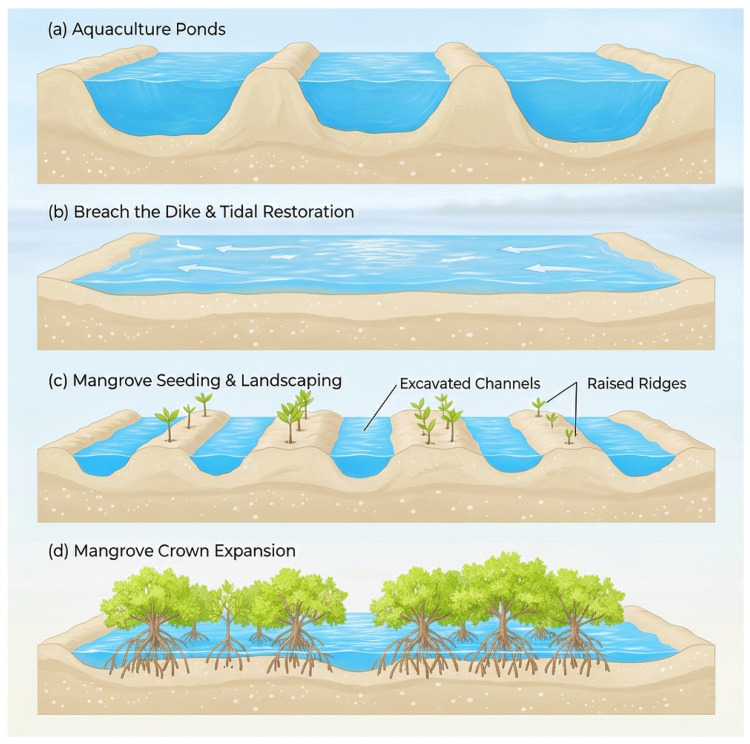
Stages of pond-to-mangrove ecological restoration. (**a**) Pre-restoration: aquaculture ponds separated by raised embankments. (**b**) Hydrological reconnection: dikes breached and tidal flow restored across pond basins. (**c**) Early restoration: tidal channels excavated, sediment ridges formed, and mangrove seedlings planted. (**d**) Late restoration: expansion of mangrove crowns and development of a continuous forest canopy.

In response to widespread mangrove degradation, large-scale restoration programs have been implemented worldwide. Two dominant strategies have emerged: tidal-flat afforestation and pond-to-mangrove restoration [15,16]. Tidal-flat afforestation, the direct planting of seedlings on intertidal flats, can increase mangrove extent rapidly but frequently poses substantial ecological trade-offs by converting essential shorebird foraging grounds, thereby reducing the abundance and diversity of mudflat-dependent species [6,17]. This strategy highlights the need to reconcile mangrove recovery with the conservation of open tidal habitats. In contrast, pond-to-mangrove restoration repurposes abandoned or low-yield aquaculture ponds as restoration substrates. By breaching embankments and reinstating tidal regimes, this approach can restore hydrological processes, promote natural sediment dynamics, and facilitate mangrove regeneration while partially retaining shallow-water features that offer supplementary roosting and foraging opportunities for waterbirds [18,19]. Owing to its potential for minimizing direct loss of intertidal flats, pond-to-mangrove conversion has been widely promoted in China’s recent national restoration initiatives, with thousands of hectares scheduled for recovery under the Mangrove Restoration Action Plan (2020–2025) (Figure 1). However, despite its rapid expansion, the ecological consequences of this increasingly adopted strategy remain poorly understood.

Although the ecological impacts of tidal-flat afforestation have been widely studied, with consistent evidence of negative effects on shorebird assemblages [6,20], the outcomes of pond-to-mangrove restoration remain much less certain. Existing evaluations have largely focused on vegetation establishment or reported only coarse bird metrics such as species richness or abundance [21,22,23], providing an incomplete understanding of community reassembly. More broadly, an increasing number of studies have advocated extending restoration assessments beyond local alpha diversity to the structure of beta diversity at broader spatial scales, because restoration actions can increase local species richness while simultaneously altering turnover and nestedness processes, thereby reshaping between-community dissimilarity and potentially promoting biotic homogenization [24,25,26]. Accordingly, the framework that partitions beta diversity into turnover and nestedness components has been widely applied to reveal mechanisms of community reassembly under habitat change and human disturbance, and to evaluate whether restoration leads to regional biotic homogenization [27,28].

Different restoration strategies may influence wetland biodiversity in contrasting ways: relative to natural regeneration, artificial restoration may simplify macrobenthos communities and promote biotic homogenization [29]; more generally, human-engineered interventions tend to homogenize mangrove wetland biota [30]. These findings raise critical questions about whether similar patterns occur in waterbird communities, particularly in terms of taxonomic beta diversity and turnover processes. In addition, mangrove expansion may reduce the extent of intertidal mudflats, thereby creating trade-offs with shorebird conservation [20]. Despite these concerns, there remains limited understanding of how pond-to-mangrove restoration affects multiple dimensions of waterbird diversity, including alpha diversity, beta diversity, and functional diversity. It is also unclear how different waterbird groups, such as egrets and shorebirds, respond to the rapid conversion of aquaculture ponds into mangroves.

Bamen Bay lies within the Qinglangang Mangrove Nature Reserve in northeastern Hainan Island and is a lagoon–estuary coastal wetland where mangroves, tidal flats, and shallow waters form a heterogeneous habitat mosaic. Mangrove dynamics have been shaped by aquaculture expansion and subsequent restoration policies. Multi-decadal remote-sensing analyses show that early mangrove loss was closely linked to conversion to aquaculture ponds and accompanied by marked landscape fragmentation. Since the mid-to-late 2010s, mangrove cover has stabilized and begun to recover following pond-to-mangrove restoration [31]. Nevertheless, regional assessments still identify Bamen Bay as an area under relatively high human pressure, and mangrove ecosystems remain mildly to moderately degraded, suggesting that long-term cumulative impacts have not been fully offset [32]. Since 2011, large-scale restoration has reconnected some ponds to tidal exchange through embankment removal, rapidly altering habitat configuration by changing the availability of shallow-water areas, open flats, and mangrove-edge habitats. Such shifts can affect waterbird roosting and foraging conditions and may be reflected in community composition as well as taxonomic and functional diversity. Evaluating multidimensional waterbird responses in this context is important for clarifying community reassembly and strengthening restoration-effectiveness assessment and adaptive management.

In this study, we used standardized bird surveys from 2021 and 2023 to evaluate the short-term ecological outcomes of pond-to-mangrove restoration in Bamen Bay. We addressed three questions. First, do restored ponds differ from nearby unrestored ponds in taxonomic α diversity and Jaccard β diversity (βSOR, βSIM, βSNE)? Second, does restoration shift functional diversity, including functional richness, evenness, and divergence? Third, do responses diverge between egrets and shorebirds, two guilds expected to differ in their sensitivity to mangrove expansion? This study is among the first to quantify early restoration responses of waterbirds across taxonomic, functional, and compositional dimensions within a BACI-like comparison of restored and unrestored ponds. We expected that tidal reconnection and early vegetation establishment would increase habitat heterogeneity and expand shallow-water and edge habitats, leading to higher α diversity and functional richness in restored ponds. We also expected detectable compositional reorganization after hydrological reinstatement, expressed mainly as higher turnover (βSIM). Finally, we expected guild divergence: egrets would respond rapidly to the expansion of mangrove–water ecotones, whereas shorebirds would show weaker or delayed responses as open mudflat habitat becomes more limited. By identifying both early ecological gains and emerging trade-offs, our results provide practical indicators for restoration evaluation and can inform bird-compatible design. In particular, they support restoration strategies that maintain a functional habitat mosaic, including retained open flats where feasible and managed shallow-water habitats within restored landscapes.

## 2. Materials and Methods

### 2.1. Study Area

**Figure 2:**
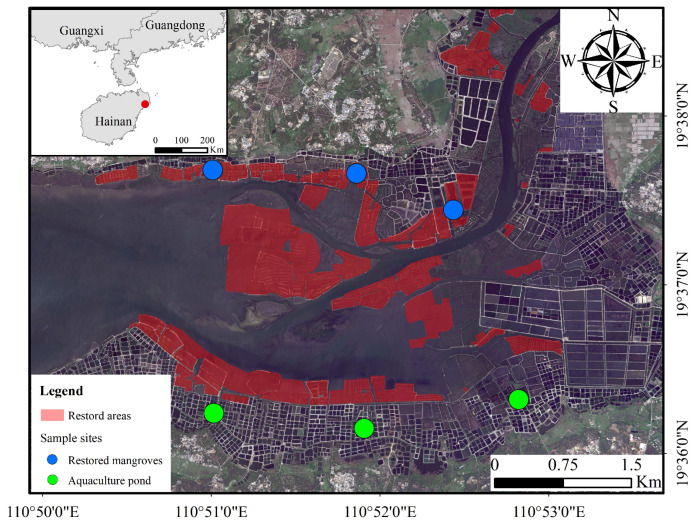
Spatial distribution of restored mangrove sites and aquaculture-pond controls within the Bamen Bay study area on Hainan Island, China.

Bamen Bay is located within the Qinglangang Mangrove Reserve in Wenchang, northeastern Hainan Island, China (110°02′–110°30′ E, 19°15′–20°09′ N) (Figure 2). The reserve contains roughly 2000 ha of mangroves and supports one of the highest mangrove species richness levels in China, dominated by Upriver orange mangrove (*Bruguiera sexangula*), Tall-stilt mangrove (*Rhizophora apiculata*), and Crabapple mangrove (*Sonneratia caseolaris*). Eight rivers, including the Wenchang River and Wenjiao River, converge into the bay, forming a lagoon–estuary complex shaped by an irregular semidiurnal tide regime. Tidal heights range from a maximum of 2.38 m to a minimum of 0.01 m, with a mean range of approximately 0.75 m. These hydrological and sedimentary conditions sustain extensive intertidal mudflats, shallow-water ponds, and tidal creeks, creating a heterogeneous coastal wetland mosaic suitable for both aquaculture and mangrove growth [33]. Bamen Bay is an important site along the East Asian–Australasian Flyway (EAAF), with approximately 128 waterbird species recorded to date. The coexistence of mangroves, mudflats, and shallow-water habitats makes Bamen Bay particularly suitable for examining how pond-to-mangrove restoration influences waterbird diversity and community composition across different functional guilds.

### 2.2. Pond-to-Mangrove Restoration in the Study Area

Over the past two decades, aquaculture ponds have covered approximately 850 ha in Bamen Bay. Prolonged intensive aquaculture and associated wastewater discharge degraded adjacent mangrove habitats and disrupted coastal ecological processes. To reverse these impacts, a long-term restoration programme was initiated in 2011, involving the decommissioning of aquaculture ponds followed by landscape-scale mangrove rehabilitation. This restoration programme was completed in 2022.

In total, 236 ha of former aquaculture ponds were reconnected to tidal influence and replanted with native mangrove species, and an additional 82 ha were restored as wetlands. Restoration focused on reinstating tidal hydrology by lowering or removing dikes and excavating tidal channels. Once suitable inundation regimes were established, native mangrove species such as Grey mangrove (*Avicennia marina*), Upriver orange mangrove (*Bruguiera sexangula*), White-flowered lumnitzera (*Lumnitzera racemosa*), and River mangrove (*Aegiceras corniculatum*) were planted at standard spacings (1–1.5 m). The restored stands have since shown high survival (>85%) and continued natural expansion, indicating effective re-establishment of mangrove structure and function.

For comparative analysis, we selected pond-to-mangrove restoration areas and active aquaculture ponds as control sites. The control ponds were located on the southern shore of Bamen Bay (Figure 2) and remained under aquaculture management during the study period. Most active aquaculture ponds in the control area were broadly similar in size, typically measuring approximately 40 × 40 m or 60 × 40 m. This spatial configuration allowed a direct comparison between restored and unrestored pond systems within the same estuarine setting, forming the basis for the BACI-style assessment of waterbird community responses to pond-to-mangrove restoration.

### 2.3. Bird Surveys

To capture seasonal variation in coastal wetland bird assemblages, we conducted surveys in March, June, September and December of 2021 and 2023, corresponding to spring migration, the breeding season, autumn migration and winter, respectively, for a total of eight survey occasions. Standardized transect surveys were not conducted in 2022, so the present study compares these two discrete survey years. We established six fixed transects (1–2 km in length) along pond embankments and adjacent mangrove edges, with three transects in restored pond-to-mangrove habitat and three in unrestored aquaculture ponds (Figure 2).

All counts were carried out by the same team of four experienced observers, working in two constant pairs. Each transect was always surveyed by the same pair to minimise observer bias [34,35]. Surveys were conducted during daylight hours between 07:00–10:30 and 15:30–18:00, avoiding heavy rain, fog, or strong winds. To maximise detectability of shorebirds, we scheduled counts within approximately ±2 h of low tide whenever possible [36]. Observers walked slowly along the transects at a speed of about 1.5–2.0 km h^−1^ and scanned ponds, mudflats and mangrove edges using binoculars (ZEISS Terra ED 10 × 42; Carl Zeiss AG, Oberkochen, Germany) and telephoto cameras (Canon EOS R5 with a Canon RF 100–500 mm telephoto lens; Canon Inc., Tokyo, Japan). For each detection, we recorded species identity, flock size, and GPS-based location by Liangbulu Outdoor Assistant (version 8.1.1-1518; Two Steps Road Technology Co., Ltd., Shenzhen, China). Birds flying high overhead without interacting with the habitat were excluded to avoid inflating abundance estimates. To reduce disturbance to waders, observers remained on existing dikes (>100 m from flocks) and paused or detoured when birds displayed alarm behavior [36,37]. When flocks moved during surveys, we recorded the maximum number of individuals seen simultaneously or counted crossing flocks only once in the direction of travel. Bird taxonomy and residency types followed the Checklist of Chinese Birds (4th edition) [38]. Conservation status was assigned according to the List of National Key Protected Wild Animals and the IUCN Red List [39].

### 2.4. Data Analysis

#### 2.4.1. Alpha and Beta Diversity

Alpha diversity (species richness, Shannon diversity, and Pielou’s evenness) was calculated for each transect in each year, season and habitat type, in order to evaluate spatial and temporal variation in local community structure. For the BACI comparisons, we then averaged the seasonal values within each combination of year and habitat to obtain an annual mean for each transect. Beta diversity was quantified using Jaccard dissimilarity based on presence–absence matrices, which is appropriate for comparing community composition when detectability may vary among heterogeneous wetland habitats. For the beta diversity analyses, each transect-season combination within a given year and habitat was treated as a separate sampling unit in the presence-absence matrix, so that Jaccard βSOR, βSIM and βSNE reflected turnover among all seasonal surveys and transects within each year and habitat. Following Baselga’s partitioning framework, total dissimilarity (βSOR) was decomposed into species turnover (βSIM) and nestedness components (βSNE) using the vegan (v2.6.4) and betapart (v1.6) packages [40,41].

#### 2.4.2. Functional Diversity

To assess functional diversity (FD), we assembled a species–trait matrix for all bird species recorded in this study. Functional trait data were obtained from the AVONET database and EltonTraits 1.0 [42,43]. We selected 13 morphological and ecological traits that represent major axes of waterbird ecology: body mass, culmen length, bill width, bill depth, tarsus length, wing length, Kipp’s distance, hand-wing index, tail length, primary lifestyle, diet type, trophic level and trophic niche. Together, these traits describe key variation in body size and locomotion, foraging mode and vertical foraging stratum, and energy acquisition pathways, and are widely used to characterise functional diversity in waterbird and wetland-bird assemblages.

After taxonomic harmonisation and matching with the community dataset, the final trait matrix used for FD calculations included 52 species. Trait completeness was checked prior to analysis; for the retained species, no missing values were present in the trait matrix used for FD computations, and thus no imputation was applied. Continuous traits were scaled by their observed range and categorical traits were coded as dummy variables. Functional distances among species were calculated using Gower’s distance as implemented in the R package FD (v1.0.12.3) via the function gowdis(), which accommodates mixed trait types [44]. No additional trait weights were applied, so all traits contributed equally to the functional distance matrix. Based on the resulting trait-distance matrix and observed species abundances, we quantified functional richness (FRic), functional evenness (FEve) and functional divergence (FDiv) using dbFD() in FD, with abundance-weighting enabled (w.abun = TRUE), Cailliez correction applied (corr = “cailliez”), and no additional standardisation within dbFD (stand.x = FALSE) [45]. To facilitate reproducibility, the full trait matrix used in this study is provided in Supplementary Table S1.

#### 2.4.3. Trend Analysis and Statistical Tests

Because standardised surveys were conducted only in 2021 and 2023, the study design captures two discrete time points rather than continuous temporal trajectories. Statistical comparisons therefore focused on between-year differences within each habitat type. Prior to hypothesis testing, alpha diversity metrics were assessed for normality using the Shapiro–Wilk test and for homogeneity of variances using Levene’s test. When parametric assumptions were met, independent-samples *t*-tests were applied; otherwise, non-parametric Mann–Whitney U tests (Wilcoxon rank–sum tests) were used. Effect sizes were reported as Cohen’s d for parametric tests and rank-biserial correlation for non-parametric tests.

For guild-level analyses, species were assigned following standard taxonomic conventions widely applied in waterbird studies. The “egret/heron” guild comprised species in the family Ardeidae. The “shorebird” guild included charadriiform waders (Charadrii and Scolopaci), including plovers and sandpipers [46,47]. These guilds are taxonomically distinct and non-overlapping in our dataset, and no ambiguous species required alternative classification.

Temporal changes in alpha, beta, and functional diversity between 2021 and 2023 were visualised using the ggplot2 package (v3.5.2) [26]. All analyses were conducted in R (version 4.3.1) [48].

## 3. Results

### 3.1. Responses of Taxonomic and Functional Diversity to Pond-to-Mangrove Restoration

**Figure 3:**
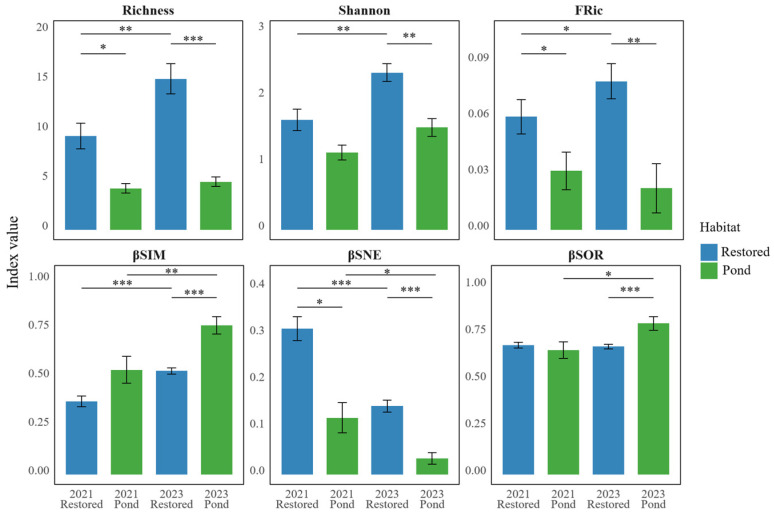
BACI comparison of bird diversity between restored mangroves and aquaculture ponds. Blue bars represent restored mangrove transects, while green bars indicate aquaculture pond transects. Horizontal lines and asterisks denote significant pairwise contrasts between years or habitats. ‘***’ indicates very strong evidence (*p* < 0.001). ‘**’ indicates strong evidence (*p* < 0.01). ‘*’ indicates moderate evidence (*p* < 0.05). βSOR indicates total dissimilarity (overall beta diversity); βSIM indicates species turnover (replacement), and βSNE indicates the nestedness component of beta diversity.

Across both survey years in our study system, taxonomic α diversity was higher in restored transects than in aquaculture ponds. Species richness was higher in restored sites than in ponds in 2021 (*p* < 0.05) and showed a further increase in 2023 (*p* < 0.001), whereas richness in ponds showed no detectable change between the two years. Shannon diversity showed a similar pattern, being higher in restored transects in 2023 than in 2021 (*p* < 0.01), while ponds showed no detectable change over time. Consistent with these patterns, Shannon diversity differed between habitats in 2023 (*p* < 0.01). Functional richness (FRic) was also higher in restored transects than in ponds in both years (2021: *p* < 0.05; 2023: *p* < 0.01) and was higher in 2023 than in 2021 in restored sites (*p* < 0.05), while remaining broadly stable in ponds. Taken together, these results highlight a contrast within our study system: overall community dissimilarity remained broadly similar across years in restored transects, whereas aquaculture ponds showed larger between-year shifts in composition (Figure 3).

Patterns of β diversity in our study system showed distinct temporal changes in the turnover and nestedness components. The turnover component (βSIM) was higher in 2023 than in 2021 in both restored mangroves (*p* < 0.001) and aquaculture ponds (*p* < 0.01). In 2023, βSIM was higher in ponds than in restored sites (*p* < 0.001). In contrast, the nestedness component (βSNE) was lower in 2023 than in 2021 in both habitats (restored: *p* < 0.001; ponds: *p* < 0.05) but remained higher in restored sites than in ponds in both 2021 (*p* < 0.05) and 2023 (*p* < 0.001). Total dissimilarity (βSOR) was broadly similar between years in restored sites but was higher in ponds in 2023 than in 2021 (*p* < 0.05), resulting in higher βSOR in ponds than in restored sites in 2023 (*p* < 0.001). Overall dissimilarity remained broadly stable in restored transects, whereas ponds showed stronger between-year shifts in composition.

### 3.2. Comparative Responses of Egrets and Shorebirds to Pond-to-Mangrove Restoration

At the functional guild level, we observed higher egret and shorebird richness in restored mangroves than in aquaculture ponds in both survey years. Egret richness was higher in restored areas (six species in 2021; nine species in 2023) than in ponds (three species in 2021; four species in 2023) (Table 1). Shorebird richness was also higher in restored mangroves than in ponds across both surveys, although the number of species recorded was lower in 2023 than in 2021 in both habitats.

**Figure 4:**
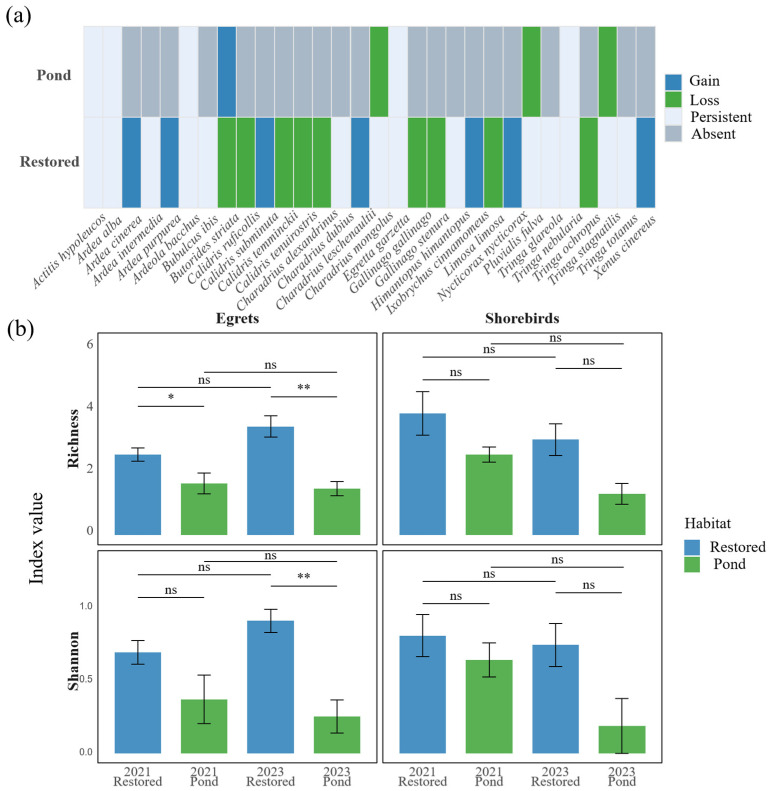
Species turnover of egrets and shorebirds in response to pond-to-mangrove restoration. (**a**) Species-level turnover of egrets and shorebirds in restored mangroves and aquaculture ponds between 2021 and 2023. Each column represents one species, and each row corresponds to a habitat type. Colours indicate species status within each habitat: Gain (present only in 2023), Loss (present only in 2021), Persistent (present in both years), and Absent (not recorded in either year). (**b**) Changes in species richness and Shannon diversity of egrets and shorebirds in restored mangroves and ponds in 2021 and 2023. Bars show mean ± SE across transects. Blue bars represent restored-mangrove transects, and green bars represent aquaculture-pond transects. Horizontal lines and asterisks indicate pairwise contrasts between years or habitats: ‘**’ indicates strong evidence (*p* < 0.01). ‘*’ indicates moderate evidence (*p* < 0.05); ‘ns’ indicates no significant difference (*p* ≥ 0.05).

At the species level, egret assemblages in restored mangroves showed turnover between years (Figure 4a). Several species recorded in 2021—Intermediate Egret (*Ardea intermedia*), Great Egret (*Ardea alba*), Chinese Pond Heron (*Ardeola bacchus*), Cattle Egret (*Bubulcus ibis*), Little Egret (*Egretta garzetta*), and Striated Heron (*Butorides striata*)—were also recorded in 2023. In addition, four heron species were newly detected in 2023: Black-crowned Night Heron (*Nycticorax nycticorax*), Cinnamon Bittern (*Ixobrychus cinnamomeus*), Grey Heron (*Ardea cinerea*), and Purple Heron (*Ardea purpurea*). Striated Heron (*Butorides striata*) was the only egret/heron species present in 2021 but not recorded in 2023. In contrast, the egret assemblage in aquaculture ponds was comparatively simple across both years, consisting mainly of Great Egret, Chinese Pond Heron, and Little Egret across both years, with Striated Heron additionally recorded in 2023 and no species recorded only in 2021.

Shorebird species composition differed between years in both habitats (Figure 4a). In restored mangroves, nine shorebird species were recorded in both years—including Wood Sandpiper (*Tringa glareola*), Marsh Sandpiper (*Tringa stagnatilis*), Common Sandpiper (*Actitis hypoleucos*), Common Redshank (*Tringa totanus*), Little Ringed Plover (*Charadrius dubius*), Pacific Golden Plover (*Pluvialis fulva*), Common Greenshank (*Tringa nebularia*), Lesser Sand Plover (*Charadrius mongolus*), and Black-winged Stilt (*Himantopus himantopus*). Three species—Terek Sandpiper (*Xenus cinereus)*, Greater Sand Plover (*Charadrius leschenaultii*), and Long-toed Stint (*Calidris subminuta*)—were recorded only in 2023. Meanwhile, eight species were recorded only in 2021: Great Knot (*Calidris tenuirostris*), Common Snipe (*Gallinago gallinago*), Kentish Plover (*Charadrius alexandrinus*), Green Sandpiper (*Tringa ochropus*), Red-necked Stint (*Calidris ruficollis*), Pin-tailed Snipe (*Gallinago stenura*), Temminck’s Stint (*Calidris temminckii*), and Black-tailed Godwit (*Limosa limosa*). In ponds, only Common Sandpiper and Common Greenshank were recorded in both surveys, while Marsh Sandpiper, Pacific Golden Plover, and Lesser Sand Plover were recorded only in 2021. Species lists are provided in Supplementary Table S2.

The diversity metrics were consistent with the species-level patterns. Egret richness was higher in restored mangroves than in ponds in 2021 (*p* < 0.05), with a larger difference in 2023 (*p* < 0.01). Within each habitat type, egret richness showed no detectable difference between years (all ns). Shannon diversity showed the same pattern (Figure 4b). In our study system, neither shorebird species richness nor Shannon diversity showed detectable differences between habitat types (restored vs. pond) or between years across the BACI contrasts.

## 4. Discussion

### 4.1. Early Ecological Responses to Pond-to-Mangrove Restoration

Overall, in our study system, pond-to-mangrove restoration was associated with higher taxonomic α diversity and functional richness in restored areas, whereas β diversity patterns differed between restored and unrestored ponds. In restored sites, total β diversity (βSOR) remained broadly similar between years, while the turnover component increased and the nestedness component declined, a pattern consistent with relatively similar composition across transects alongside species replacement. In contrast, aquaculture ponds showed higher total β diversity and higher turnover in 2023 than in 2021, together with lower nestedness, consistent with stronger between-year shifts in composition rather than stable nested subsets. This combination of increased turnover and decreased nestedness is broadly consistent with patterns reported in some mangrove restoration studies, where anthropogenic interventions have been linked to changes in compositional similarity and turnover over time [30].

During the early stages of ecological restoration, actions such as breaching embankments, excavating tidal channels, and planting mangrove seedlings can modify the simplified structure of aquaculture ponds and create a finer-scale mosaic of intertidal flats, young mangrove stands and shallow-water areas. In Bamen Bay, these structural changes could be consistent with the higher α diversity observed in restored ponds. More heterogeneous physical structure may increase the range of resources and microhabitats, which may facilitate the coexistence of species with contrasting foraging strategies. This interpretation aligns with the habitat heterogeneity hypothesis, which proposes that structurally complex environments can support more species through niche diversification [49,50,51,52]. Although we did not quantify habitat or prey variables such as mudflat extent, vegetation cover, water depth or prey abundance along transects, the diversity patterns observed in this case study are in line with mechanisms proposed in previous restoration studies [53,54,55,56].

Restored hydrological connectivity has also been reported in other systems to increase the abundance of benthic invertebrates, fish, and crustaceans [57,58], and similar processes could also be consistent with the higher α diversity and functional richness observed here. Higher functional richness reflects a broader range of trait space within the assemblage, which is consistent with the early occurrence of species occupying distinct ecological niches. Comparable responses have been reported in other large-scale pond-to-mangrove projects, including examples from the Philippines [46,59].

Overall, in our study system, restored and unrestored areas may play complementary ecological roles during the early restoration stage. Restored ponds were associated with higher taxonomic and functional diversity, whereas unrestored ponds may contribute to regional diversity through the assemblages recorded during surveys. Further work that combines bird monitoring with detailed measurements of habitat structure and prey availability will be needed to evaluate these mechanisms more directly.

### 4.2. Guild-Specific Responses to Pond-to-Mangrove Restoration

In our study system, herons and shorebirds showed different patterns in relation to pond-to-mangrove restoration. For herons, species richness and Shannon diversity were higher in restored areas than in aquaculture ponds in both years, and the habitat difference became more pronounced in 2023. This pattern is consistent with the idea that early-stage restoration-related habitat features may favor herons. By contrast, the number of shorebird species recorded in 2023 was lower than in 2021, and functional richness also declined. Taken together, these findings are consistent with an “asymmetric” early-stage restoration response: increases in overall α diversity and functional richness in restored areas were not evenly reflected across guilds.

The comparatively positive heron patterns may relate to their ecological flexibility and their frequent use of shallow-water habitats and edges that can emerge during early mangrove establishment. Many heron species recorded in this study, such as Little egret (*Egretta garzetta*) and Great egret (*Ardea alba*), are widespread generalists that forage opportunistically across wetlands [46]. In the restored areas, measures such as reconnecting tidal flow, excavating tidal channels, and planting mangrove seedlings can create shallow-water depressions, tidal creeks, and forest edges. These habitat features could increase foraging opportunities and roosting sites, which may be consistent with the higher heron richness and Shannon diversity observed in restored transects. Similar associations have been reported in other wetland restoration studies, where improved hydrological connectivity and increased structural complexity are linked to rapid increases in habitat use by generalist waterbirds [55,60,61]. However, because prey availability and microhabitat structure were not directly measured in this study, these mechanisms should be interpreted as plausible explanations based on existing literature and local landscape characteristics, rather than as directly verified causal relationships [62].

In contrast, the response of shorebirds was more complex. Although α diversity metrics did not show detectable differences in this case study, we recorded lower shorebird species richness and functional richness in 2023 than in 2021. The decline coincided with the progressive replacement of exposed mudflat areas by mangroves and shallow-water habitats, which is consistent with the ecological habits of shorebirds that heavily rely on open, unvegetated tidal flats [20,63]. During the early stages of restoration, some exposed mudflats remained in the restored areas, which could have buffered short-term impacts on shorebird occurrence. Nevertheless, potential time-lagged effects of ongoing habitat change on shorebirds warrant continued monitoring and longer-term assessment.

Together, the heron-favored patterns and the limited or declining shorebird patterns are consistent with an “asymmetric effect” during early pond-to-mangrove restoration. While restoration may enhance overall diversity metrics in this case study, the apparent benefits can differ among functional groups. This “mangrove expansion–mudflat restriction” trade-off has been noted in multiple coastal restoration contexts. For example, after mangrove afforestation in the Gulf of Thailand, the bird community shifted from being dominated by shorebirds to one dominated by forest-edge species such as herons. In contrast, at the Tamsui River estuary in Taiwan, the removal of some mangroves and the restoration of mudflats significantly promoted the return of migratory shorebirds [64]. These cases, together with our study, reveal a key insight: pond-to-mangrove restoration improves overall waterbird diversity and habitat suitability for generalist species like herons but inevitably reshapes habitat patterns, posing challenges for highly specialized shorebirds that depend on open mudflats. Therefore, when planning and evaluating such restoration projects, it is crucial to acknowledge the differential distribution of ecological benefits and consider mitigating the potential negative impacts on specialist species through spatial configurations, such as preserving or restoring portions of mudflat habitats at the regional scale [58,63].

### 4.3. Implications for Ecological Restoration and Management

Our findings highlight both the beneficial effects and potential risks with pond-to-mangrove restoration, underscoring the need to refine current restoration goals and implementation strategies. While restoration clearly enhances habitat suitability for herons and other generalist waterbirds, it simultaneously reduces the extent of open mudflats that are essential for shorebirds [20,63]. This emerging trade-off suggests that mangrove expansion and shorebird conservation may not always be compatible without deliberate planning.

First, restoration should not solely prioritize maximizing mangrove cover but should explicitly consider the ecological requirements of different bird groups. In many projects, emphasis on afforestation targets has overshadowed the ecological role of open tidal flats and shallow-water foraging zones. Exposed mudflats provide irreplaceable foraging opportunities for shorebirds [63,65], and their complete conversion to mangrove stands inevitably constrains mudflat specialists. To mitigate this, a proportion of open mudflat and shallow-water areas should be intentionally retained as functional components of the restored system [63]. The establishment of semi-open transition zones between mangroves and tidal flats may help maintain structural heterogeneity and support multiple bird guilds.

Second, our findings highlight that restoration objectives should reflect the multifunctional nature of the coastal wetlands, as prior research has shown that focusing solely on single metrics, such as vegetation cover, does not guarantee ecosystem recovery [58,66,67]. In Bamen Bay, restored ponds already supported higher taxonomic α diversity and functional richness than aquaculture ponds, yet these gains were accompanied by a gradual reduction in open mudflat area and no clear increase in shorebird diversity. Similar mismatches between structural recovery and faunal or functional responses have been reported in global syntheses of wetland restoration, where vegetation often recovers more rapidly than ecosystem processes or animal communities [68,69]. Evidence from Chinese coastal wetlands likewise shows that assessments based solely on plant communities can overestimate restoration success if hydrology and animal assemblages are not explicitly considered [70]. Recent work on mangroves as Nature-based Solutions further emphasises that restoration planning should integrate biodiversity and ecosystem-service goals rather than focusing only on increasing mangrove area [13,71]. Building on this evidence and our results, future assessment frameworks for pond-to-mangrove projects should incorporate multiple ecological dimensions, including bird diversity, the diversity and spatial configuration of habitat types, and the degree of hydrological restoration, so as to capture both early ecological gains and emerging trade-offs [72,73].

Finally, the ecological and management value of aquaculture ponds warrants greater recognition. Rather than eliminating all ponds during restoration, selective retention and adaptive management can provide complementary habitats for waterbirds while supporting socio-economic needs [74]. Certain ponds can be converted into managed shallow-water habitats through water-level regulation, while others can be maintained as ecological or low-impact aquaculture systems. Incorporating aquaculture ponds into the broader geomorphological and ecological planning of mangrove wetlands can enhance overall landscape diversity and sustain regional waterbird assemblages.

### 4.4. Research Limitations and Future Directions

This study provides one of the first empirical assessments of pond-to-mangrove restoration effects on waterbird assemblages in China; however, several limitations need to be acknowledged. First, our data capture the initial ecological response during the early post-restoration years. Early responses often reflect short-term habitat restructuring rather than the longer-term successional trajectories that shape community assembly. As mangrove canopies mature and intertidal surfaces contract, the relative suitability of restored areas for different bird guilds may change. Because only two discrete sampling years were available, the temporal patterns observed here should be interpreted cautiously. Long-term monitoring will therefore be essential for determining whether the early benefits observed for herons persist and whether shorebird responses exhibit delayed declines [75].

Second, our inference is constrained by limited spatial replication. We sampled only three transects per habitat, all located within a single bay. This small sample size reduces statistical power and increases the risk of Type II error, especially for guild-level comparisons where effect sizes may be modest [76]. In addition, transects within each habitat lie within the same pond–mangrove complex and are not fully independent, so the study is best regarded as a BACI-style case study rather than a fully replicated landscape experiment. Potential spatial autocorrelation and pseudoreplication should therefore be considered when generalizing these results beyond Bamen Bay. Moreover, we did not implement small-sample inference approaches (e.g., transect-level permutation tests or bootstrapped effect-size confidence intervals) in the current analyses. Applying these methods, together with increased spatial replication, would provide more robust inference under limited replication. Increasing the number of sites across ponds with contrasting management histories, hydrological settings and restoration intensities would strengthen the generality of future inferences.

Third, our study relied on field-based bird surveys and lacked transect-level measurements of habitat and prey (e.g., mudflat extent, vegetation cover, water depth, or prey abundance), and we did not collect the distance or repeat-visit information required for formal detectability modelling. Because β diversity was calculated using presence–absence Jaccard indices, habitat-related variation in detectability could bias comparisons: structurally complex restored sites may increase detection for conspicuous or edge-associated species (e.g., herons) while reducing detection for more cryptic taxa, which could inflate α diversity estimates and increase apparent turnover (βSIM) when species are recorded inconsistently across surveys; functional metrics may likewise be skewed toward more detectable trait profiles [77,78]. Moreover, unmeasured transect-level differences (e.g., distance to the main tidal channel, pond size, management status, or local mangrove cover) may provide alternative explanations for some among-transect variation.

Finally, treating season as an independent sampling unit in the β diversity analyses may emphasise short-term compositional dynamics, such that βSIM (and potentially βSOR) is higher than estimates based on annually pooled transect data, where seasonal variation is averaged and year-level species pools are more complete [79,80]. Accordingly, our results should be interpreted as conditional on the standardised survey protocol and the temporal resolution adopted in this study. Future work integrating bird monitoring with habitat and prey measurements, tidal hydrodynamics, and remote-sensing–derived habitat configuration metrics, together with explicit detection modelling and sensitivity analyses comparing seasonal versus annually pooled β diversity, would strengthen inference regarding restoration-driven community change [81].

Finally, a promising direction for future work is to evaluate biodiversity outcomes across multiple taxa. Because pond-to-mangrove restoration simultaneously alters vegetation, hydrology, and benthic communities, multi-taxon approaches (e.g., macrobenthos, fish, insects) may reveal whether birds respond earlier or differently relative to other components of the ecosystem, and whether restoration produces coordinated or decoupled biodiversity trajectories [82,83,84].

Together, these future research directions highlight the need for integrated, long-term, and multi-taxon approaches to fully understand the ecological consequences of pond-to-mangrove restoration across scales.

## 5. Conclusions

This study provides one of the first empirical assessments of pond-to-mangrove restoration on waterbird communities in China. Early-stage restoration increased α and functional diversity, with herons and other generalist species benefiting from expanded shallow-water and mangrove-edge habitats. However, shorebirds, dependent on mudflats, showed a limited immediate response. These findings highlight that pond-to-mangrove restoration is a multidimensional transformation that may produce both ecological opportunities and trade-offs. Programs prioritizing mangrove cover alone may overlook the value of open tidal flats and aquaculture ponds. Maintaining a mosaic of habitat types, including mangroves, shallow-water areas, and retained mudflats, is crucial for supporting diverse waterbird guilds. Future restoration efforts should integrate hydrological design, habitat configuration, and adaptive management, with long-term monitoring across multiple taxa to assess the sustainability of early biodiversity gains. Balancing mangrove recovery with waterbird conservation requires a landscape-level approach that recognizes the interdependence of mangroves, tidal flats, and aquaculture wetlands.

## Figures and Tables

**Table 1:** Presence–absence of shorebird and egret species recorded in restored ponds and aquaculture ponds in 2021 and 2023.

Waterbird Groups	Scientific Name	Year 2021		Year 2023	
		Restoration Zone	Aquaculture Ponds	Restoration Zone	Aquaculture Ponds
Shorebirds	*Actitis hypoleucos*	√	√	√	√
	*Calidris ruficollis*	√	-	-	-
	*Calidris subminuta*	-	-	√	-
	*Calidris temminckii*	√	-	-	-
	*Calidris tenuirostris*	√	-	-	-
	*Charadrius alexandrinus*	√	-	-	-
	*Charadrius dubius*	√	-	√	-
	*Charadrius leschenaultii*	-	-	√	-
	*Charadrius mongolus*	√	√	√	-
	*Gallinago gallinago*	√	-	-	-
	*Gallinago stenura*	√	-	-	-
	*Himantopus himantopus*	√	-	√	-
	*Limosa limosa*	√	-	-	-
	*Pluvialis fulva*	√	√	√	-
	*Tringa glareola*	√	-	√	-
	*Tringa nebularia*	√	√	√	√
	*Tringa ochropus*	√	-	-	-
	*Tringa stagnatilis*	√	√	√	-
	*Tringa totanus*	√	-	√	-
	*Xenus cinereus*	-	-	√	-
Egrets	*Ardea alba*	√	√	√	√
	*Ardea cinerea*	-	-	√	-
	*Ardea intermedia*	√	-	√	-
	*Ardea purpurea*	-	-	√	-
	*Ardeola bacchus*	√	√	√	√
	*Bubulcus ibis*	√	-	√	-
	*Butorides striata*	√	-	-	√
	*Egretta garzetta*	√	√	√	√
	*Ixobrychus cinnamomeus*	-	-	√	-
	*Nycticorax nycticorax*	-	-	√	-

Presence is indicated by √; “-” indicates absence.

## Data Availability

All data supporting the findings of this study are available within the article and its Supplementary Information files.

## Supplementary Materials

The following supporting information can be downloaded at: https://www.mdpi.com/article/10.3390/ani16020299/s1, Table S1: Species-level functional trait matrix; Table S2: Species abundance in the restoration zone and aquaculture ponds in 2021 and 2023.
