# Supplementary material for: Ecological Trade-Offs Between Mangrove Expansion and Waterbird Diversity: Guild-Specific Responses to Pond-to-Mangrove Restoration
Source: Animals (Basel). 2026 Jan 19;16(2):299. doi: 10.3390/ani16020299 (PMC12837314; doi:10.3390/ani16020299)
Supplement: Supplementary file 1 [file animals-16-00299-s001.zip › animals-4071963-supplementary.pdf]

Supplementary

Table S1. Species-level functional trait matrix

Species	body mass	Beak.Length_Culmen	Tarsus.Length	Beak.Width	Beak.Depth	Wing.Length	Kipps.Distance	Hand-Wing.Index	Tail.Length	diet type	Trophic.Level	Trophic.Niche	Primary.Lifestyle
Acridotheres cristatellus	116.31	27.1	38.1	6.1	8.1	134	32.1	24.1	81.2	Omnivorous	Omnivore	Omnivore	Generalist
Actitis hypoleucos	48	30.1	22.4	3.1	4	109.9	51.1	48	57.4	Insectivorous	Carnivore	Aquatic predator	Terrestrial
Alcedo atthis	31.09	42.2	9.9	5.7	7.4	73.3	19.3	26.9	32.3	Carnivorous	Carnivore	Aquatic predator	Insessorial
Amaurornis phoenicurus	180	37.1	50.8	4.4	8.8	147	33.7	23.2	57.6	Omnivorous	Carnivore	Aquatic predator	Terrestrial
Ardea alba	871.33	126.3	141.6	12	18.1	378.8	120.7	32.1	140.5	Insectivorous and carnivorous	Carnivore	Aquatic predator	Terrestrial
Ardea cinerea	1443	138.9	137.9	18.7	25.4	441	152.7	34.6	169.1	Carnivorous and insectivorous	Carnivore	Aquatic predator	Terrestrial
Ardea intermedia	458.83	90.4	104.2	10.5	14.1	303.4	98.4	32.7	117.8	Carnivorous and insectivorous	Carnivore	Aquatic predator	Terrestrial
Ardea purpurea	1064.48	125.9	117.5	13.6	19.6	349.7	118.2	33.9	113.2	Carnivorous and insectivorous	Carnivore	Aquatic predator	Terrestrial
Ardeola bacchus	304.89	72.9	56.2	9.2	12.2	213	56.6	26.8	73.4	Carnivorous and insectivorous	Carnivore	Aquatic predator	Terrestrial
Bubulcus ibis	365.95	63.3	76.8	8.6	11.5	244.4	73	29.9	90.9	Insectivorous and carnivorous	Carnivore	Omnivore	Terrestrial
Butorides striata	201.5	69.2	49.7	9.1	11.2	175.9	53.2	29.8	63.6	Carnivorous and insectivorous	Carnivore	Aquatic predator	Terrestrial

Calidris ruficollis	26.79	19	18.8	2.4	3.3	99.5	54.2	54.5	43.5	Insectivorous	Carnivore	Aquatic predator	Terrestrial
Calidris subminuta	30.2	20.1	21.5	2.5	3.4	88.2	43.9	49.4	35.8	Insectivorous	Carnivore	Aquatic predator	Terrestrial
Calidris temminckii	23	19	17.8	2.3	3.1	96.7	47.9	50	46.2	Insectivorous	Carnivore	Aquatic predator	Terrestrial
Calidris tenuirostris	192	45	34.4	4.5	6.2	175.6	101.2	57.3	63.8	Insectivorous	Carnivore	Aquatic predator	Terrestrial
Ceryle rudis	84.37	64.2	11	9.1	11.6	136.2	47.5	34.5	75.7	Carnivorous and insectivorous	Carnivore	Aquatic predator	Insessorial
Charadrius alexandrinus	42.3	16.3	26.5	3.1	3.1	106.2	59.2	55	44.2	Insectivorous and carnivorous	Carnivore	Aquatic predator	Terrestrial
Charadrius dubius	38.7	16.1	23.2	2.6	3.1	114.8	57.2	51	55.6	Insectivorous	Carnivore	Aquatic predator	Terrestrial
Charadrius leschenaultii	74.8	26.5	36.5	4.2	5.4	136.4	72	53.1	49.8	Insectivorous	Carnivore	Aquatic predator	Terrestrial
Charadrius mongolus	64	18	29.8	4	4.2	128.8	75.8	58.5	52.8	Insectivorous and carnivorous	Carnivore	Aquatic predator	Terrestrial
Egretta garzetta	312	94.6	98.8	8.9	11.3	252.5	82.8	33.1	94.2	Carnivorous and insectivorous	Carnivore	Aquatic predator	Terrestrial
Gallinago gallinago	112.94	69.7	30	4.6	6	127.9	56.6	44.3	54	Insectivorous	Carnivore	Aquatic predator	Terrestrial
Gallinago stenura	113	65.7	30.5	5.2	6.9	126.8	59.3	46.5	44	Insectivorous	Carnivore	Aquatic predator	Terrestrial
Gallinula chloropus	339.63	33.9	45.9	4.3	8.1	165.5	53	32.2	69	Omnivorous	Omnivore	Omnivore	Terrestrial

Halcyon pileata	83.99	65.9	14.2	14.1	14.8	124	32.7	25.7	80.3	Carnivorous and insectivorous	Carnivore	Invertivore	Insessorial
Halcyon smyrnensis	91.4	59.8	15	13.7	14.3	113.2	24.5	21.7	78.3	Carnivorous and insectivorous	Carnivore	Omnivore	Insessorial
Himantopus himantopus	176.82	65.7	110.8	4.5	4.7	223.8	117.2	52.2	74	Carnivorous and insectivorous	Carnivore	Aquatic predator	Terrestrial
Ixobrychus cinnamomeus	126.49	58.7	46.9	6.6	10.6	144.3	36.1	25.6	40.4	Carnivorous and insectivorous	Carnivore	Aquatic predator	Terrestrial
Limosa limosa	288.37	113.5	78	6	10	215.6	110.2	51.5	78	Insectivorous	Carnivore	Aquatic predator	Terrestrial
Nycticorax nycticorax	810	80.5	69.2	12.1	18.6	285.7	92.7	33.1	95.8	Carnivorous and insectivorous	Carnivore	Aquatic predator	Terrestrial
Pluvialis fulva	134.9	25.2	40.2	4.2	5.2	159	94.8	59.3	58.4	Insectivorous and carnivorous	Carnivore	Omnivore	Terrestrial
Spatula querquedula	325.6	39.2	26	13.8	12.5	188.2	105.6	56.1	64.2	Omnivorous	Omnivore	Herbivore aquatic	Aquatic
Tachybaptus ruficollis	169.43	23.9	35.4	4.4	6.7	100	31.6	32.2	29.1	Carnivorous and insectivorous	Carnivore	Aquatic predator	Aquatic
Tringa glareola	62.05	32.5	35.1	2.9	3.9	124	60.2	48.4	49.2	Insectivorous	Carnivore	Aquatic predator	Terrestrial
Tringa nebularia	187	63.6	58	4.1	6	185.4	96.9	52.1	74.8	Insectivorous	Carnivore	Aquatic predator	Terrestrial
Tringa ochropus	71.4	37.4	32.9	2.7	4.1	137.8	63.2	45.8	59.5	Insectivorous	Carnivore	Aquatic predator	Terrestrial
Tringa stagnatilis	77.5	42.5	49.9	3	3.6	133.4	68.9	51.7	56.2	Insectivorous	Carnivore	Aquatic predator	Terrestrial

Tringa totanus	129	45.1	47.3	3.4	4.9	156.9	74.1	47.2	64.1	Insectivorous	Carnivore	Aquatic predator	Terrestrial
Xenus cinereus	78.8	46.8	28.2	4	4.5	127.4	70	54.6	55	Insectivorous	Carnivore	Aquatic predator	Terrestrial
Circus spilonotus	558.78	38.3	86.3	12.1	15.9	407.2	194.2	47.7	241.2	Carnivorous and scavenging	Carnivore	Vertivore	Aerial
Pycnonotus aurigaster	44.33	19.4	21.9	4.1	4.8	91.8	14	15.5	82.4	Omnivorous	Herbivore	Omnivore	Insessorial
Motacilla alba	23.93	16.1	23.4	3.4	3.5	87	27.4	31.4	86.8	Insectivorous	Carnivore	Invertivore	Terrestrial
Pycnonotus sinensis	34.2	17.9	21.6	4.4	4.9	86.7	17.8	20.8	82.1	Omnivorous	Herbivore	Frugivore	Insessorial
Upupa epops	66.93	55.8	21.1	5.5	6.3	142.3	30.5	21.4	96.9	Insectivorous	Carnivore	Invertivore	Terrestrial
Saxicola stejnegeri	14.1	15.3	21.9	3.5	3.4	67.3	14.0	20.8	48.3	Insectivorous	Carnivore	Invertivore	Insessorial
Centropus sinensis	280.7	46.6	55.6	9.9	16.3	212.4	30.9	14.6	260.9	Insectivorous and carnivorous	Carnivore	Vertivore	Terrestrial
Nisaetus cirrhatus	1475.12	49.9	97.7	12.6	21.7	414.6	115.8	27.9	289.8	Carnivorous	Carnivore	Vertivore	Insessorial
Phylloscopus fuscatus	8.74	11.9	21.4	2.5	2.6	62.3	12.1	19.1	53.4	Insectivorous	Carnivore	Invertivore	Insessorial
Elanus caeruleus	259.76	30.9	32.1	9.2	11.3	286.3	132.1	45.2	127.7	Carnivorous and insectivorous	Carnivore	Vertivore	Aerial
Dicrurus macrocercus	48.3	25.4	22.2	8.4	8.4	141	39.2	27.8	145.6	Insectivorous	Carnivore	Invertivore	Insessorial
Gracupica nigricollis	156.92	32.4	41.7	6	7.9	158.1	41.3	26.2	95.4	Omnivorous	Carnivore	Invertivore	Insessorial

Cinnyris jugularis	8.99	20.7	14.5	3.3	2.5	53.4	8.8	17.7	35.2	Florivorous	Omnivore	Omnivore	Insessorial
Sturnia sinensis	61	20.4	25.6	5.1	5.1	101.8	32.6	32.3	57.5	Omnivorous	Carnivore	Invertivore	Generalist
Hirundo rustica	17.91	12.4	11.7	4.5	2.7	115.8	62.3	52.6	71.4	Insectivorous	Carnivore	Invertivore	Aerial
Merops orientalis	14.8	29.4	11.3	5.4	4.8	91.2	32.2	35.4	69.8	Insectivorous	Carnivore	Invertivore	Insessorial
Phaenicophaeus tristis	117	35.6	32.8	9.4	12.6	165.7	27	16	333.3	Insectivorous	Carnivore	Invertivore	Insessorial
Copsychus saularis	36	23.2	29.5	5.4	5.8	97.5	18.7	19.1	84	Insectivorous	Carnivore	Invertivore	Terrestrial
Cuculus micropterus	88.75	30.3	19.5	8.4	9.3	196.3	87.9	45.1	146.1	Insectivorous	Carnivore	Invertivore	Insessorial
Apus affinis	22.57	8.5	10.3	5.6	2.8	126.5	81.7	66	41.5	Insectivorous	Carnivore	Invertivore	Aerial
Centropus bengalensis	148.91	28.2	39.3	6.6	11	154.6	29.6	18.6	185.2	Insectivorous	Carnivore	Invertivore	Terrestrial
Spilopelia chinensis	159	19.9	19.9	3.5	3.5	138.8	42	30.3	127.3	Omnivorous	Herbivore	Granivore	Terrestrial
Lanius schach	51.51	21.8	29.2	6.8	9.2	92.4	18.6	20.3	123.3	Insectivorous and carnivorous	Carnivore	Invertivore	Insessorial

Table S2. Species abundance in the restoration zone and aquaculture ponds in 2021 and 2023.

Scientific name	**Year 2021**		**Year 2023**		**Total**
	Restoration zone	Aquaculture ponds	Restoration zone	Aquaculture ponds	
Acridotheres cristatellus	32		111	7	150
Actitis hypoleucos	27	1	46	5	79
Alcedo atthis	11		5		16
Amaurornis phoenicurus	1		2		3
Ardea alba	23	1	49	3	76
Ardea cinerea			2		2
Ardea intermedia	1		11		12
Ardea purpurea			1		1
Ardeola bacchus	62	9	38	19	128
Bubulcus ibis	10		36		46
Butorides striata	2			2	4
Calidris ruficollis	3				3
Calidris subminuta			18		18
Calidris temminckii	3				3
Calidris tenuirostris	1				1
Ceryle rudis	29		16		45
Charadrius alexandrinus	2				2
Charadrius dubius	16		1		17
Charadrius leschenaultii			2		2
Charadrius mongolus	60	50	8		118
Egretta garzetta	192	24	170	16	402
Gallinago gallinago	13				13
Gallinago stenura	1				1
Gallinula chloropus	5		5		10
Halcyon pileata	1				1

Halcyon smyrnensis	18		24	2	44
Himantopus himantopus	1		104		105
Ixobrychus cinnamomeus			4		4
Limosa limosa	1				1
Nycticorax nycticorax			3		3
Pluvialis fulva	143	6	19		168
Spatula querquedula			3		3
Tachybaptus ruficollis			2		2
Tringa glareola	37		5		42
Tringa nebularia	94	6	36	6	142
Tringa ochropus	1				1
Tringa stagnatilis	4	38	16		58
Tringa totanus	1		35		36
Xenus cinereus			3		3
Circus spilonotus			1		1
Pycnonotus aurigaster			2		2
Motacilla alba		4	3		7
Pycnonotus sinensis			7		7
Upupa epops	2		5		7
Saxicola stejnegeri				1	1
Centropus sinensis	3		15	1	19
Nisaetus cirrhatus			4		4
Phylloscopus fuscatus			8	1	9
Elanus caeruleus			3		3
Dicrurus macrocercus	4		21		25
Gracupica nigricollis	3		10		13
Cinnyris jugularis			1		1
Sturnia sinensis			131	36	167
Hirundo rustica			87	21	108

Merops orientalis	6		12		18
Phaenicophaeus tristis			1		1
Copsychus saularis	2		15	5	22
Cuculus micropterus			4		4
Apus affinis			20		20
Centropus bengalensis			1		1
Spilopelia chinensis	21		3		24
Lanius schach	5	1	12	1	19
Total	841	140	1141	126	2248
